# Characterization and Interrelations of One-Carbon Metabolites in Tissues, Erythrocytes, and Plasma in Mice with Dietary Induced Folate Deficiency

**DOI:** 10.3390/nu9050462

**Published:** 2017-05-05

**Authors:** Markus Kopp, Rosalie Morisset, Michael Rychlik

**Affiliations:** 1 Chair of Analytical Food Chemistry, Technical University of Munich, Alte Akademie 10, Freising D-85354, Germany; markus.kopp@tum.de; 2 Institute for Food & Health (Z I E L), Technical University of Munich, Weihenstephaner Berg 1, Freising D-85354, Germany; rosalie.morisset@tum.de; 3 Chair of Nutritional Physiology, Technical University of Munich, Gregor-Mendel-Straße 2, Freising D-85354, Germany; 4 Centre for Nutrition and Food Sciences, Queensland Alliance for Agriculture and Food Innovation (QAAFI), University of Queensland, Brisbane, QLD 4072, Australia

**Keywords:** folate metabolism, methionine metabolism, *S*-adenosylhomocysteine, *S*-adenosylmethionine, folate deficiency, homocysteine

## Abstract

Studies on one-carbon metabolism for the assessment of folate deficiency have focused on either metabolites of folate metabolism or methionine cycle. To bridge the gap between deficiency markers in these pathways we designed a dietary induced folate deficiency study using male C57BL/6N mice. After weaning (3 weeks) mice were fed a defined control diet (1 week) before being fed a folate deficient diet (*n* = 6 mice) and the control diet (*n* = 6 mice) for 12 additional weeks. Thereafter, we determined total homocysteine in plasma and folate in erythrocytes as well as *S*-adenosylmethionine, *S*-adenosylhomocysteine, and six folate vitamers in tissues including 5-methyltetrahydrofolate, 5-formyltetrahydrofolate, 5,10-methenyltetrahydrofolate, tetrahydrofolate, 10-formylfolic acid, and folic acid by means of stable isotope dilution assays coupled with liquid chromatography tandem mass spectrometry. In all organs, except heart (mainly 5-mehtyltetrahydrofolate), tetrahydrofolate constitutes the main vitamer. Moreover, in liver tetrahydrofolate was most abundant followed by 5-methyltetrahydrofolate (heart: tetrahydrofolate), 5-formyltetrahydrofolate, and 5,10-methenyltetrahydrofolate. Because of the significant decrease (*p* < 0.05) of folate status and *S*-adenosylmethionine/*S*-adenosylhomocysteine ratio accompanied with increasing *S*-adenosylhomocysteine (*p* < 0.05), hepatocytes are most susceptible to folate deficiency. To the best of our knowledge, we herein present the first method for simultaneous quantitation of eight metabolites for both folate and methionine cycle in one tissue sample, tHcy in plasma, and erythrocyte folate to shed light on physiological interrelations of one-carbon metabolism.

## 1. Introduction

Folate deficiency is considered to be a potential risk factor for neural tube defects innewborns [1,2], cardiovascular diseases [3], Alzheimer’s disease [4,5], and certain forms ofcancer [6,7]. Folate deficiency studies have been conducted predominantly on rats [8,9,10,11], as their relatively higher tissue weight and the use of whole organs for folate analysis does not necessarily require sensitive methods. Another advantage of rats is the fact that some strains (e.g., F344) [12] are more susceptible to folate/methyl-deficient diets and hypomethylation than the nowadays most commonly used mice (e.g., C57BL/6J mice) [13]. Modern stable isotope dilution assays coupled with liquid chromatography tandem mass spectrometry (SIDA-LC-MS/MS) methods are used for the determination of *S*-adenosylmethionine (AdoMet) and *S*-adenosylhomocysteine (AdoHcy) in mouse tissue [14,15]. Unfortunately, reference data on AdoMet and AdoHcy in mouse tissue is largely based on results obtained from methods using unspecific liquid chromatography coupled with UV detection (LC-UV) [16,17] and data availability on the distribution of AdoMet or AdoHcy in tissue is poor [14,15,16,17,18]. The large variety of methods, animals, and diets used for assaying either folates or catabolites of the methionine cycle in separate studies does not allow any correlation to be determined between these metabolic pathways. Recently, we developed and validated the first miniaturized stable isotope dilution assay for the determination of six folate vitamers including tetrahydrofolate (H_4_folate), 5-methyltetrahydrofolate (5-CH_3_-H_4_folate), 5-formyltetrahydrofolate (5-HCO-H_4_folate), and 5,10-methenyltetrahydrofolate (5,10-CH^+^-H_4_folate) as well as AdoMet and AdoHcy to characterize both pathways simultaneously in one tissue sample by SIDA-LC-MS/MS analysis [19]. Although recent studies investigated the effect of folate deficiency on, for example, carcinogenesis or DNA-methylation, literature and studies still lack detailed and combined screenings of folate vitamers and metabolites of the methionine cycle in addition to these experiments. 

For the detailed metabolic characterization of dietary induced folate deficiency, the following organs and biomarkers have to be considered for analysis. Liver and kidney play a crucial role in vitamin homeostasis [20], folate storage [21], and creatine synthesis [22,23,24]. Due to their systemic linkage to the latter organs, brain and heart, representing, at least in part, central nervous system (CNS) and circulation, have to be considered for folate deficiency–disease interrelations affecting those metabolic systems. To characterize metabolic changes during depletion of folate stores and decreased methylation capacity in selected tissues, the tissue levels of *S*-adenosylmethionine (AdoMet) and *S*-adenosylhomocysteine (AdoHcy), which are directly involved in the methionine circle, have to be examined. AdoMet is a ubiquitous donor of methyl groups and is directly involved in the methylation of DNA, proteins, phospholipids, and neurotransmitters [25]. Increased concentrations of AdoHcy inhibit methionine synthase and betaine homocysteine methyltransferase (BHMT) and activate methylenetetrahydrofolate reductase along with cystathionine-β-synthase [26]. Total homocysteine in plasma and erythrocyte folate are used as biomarkers for folate deficiency. Erythrocyte folate status represents long-term supply because red blood cells retain folates during a period of approximately four months [27] as a possible result of hemoglobin binding [28]. Because of the small blood specimens (<200 µL) obtained from mice we adopted the method published recently by Mönch et al. [29] using 50 mg of freeze-dried erythrocytes for folate status assessment. Total homocysteine in plasma (tHcy) represents a risk factor for arteriosclerotic diseases in coronary, cerebral, and peripheral vessels as well as arterial and venous thromboembolism [30,31,32] and might be used as a biomarker for impaired methylation capacity and elevated intracellular AdoHcy levels [13].

**Figure 1:**
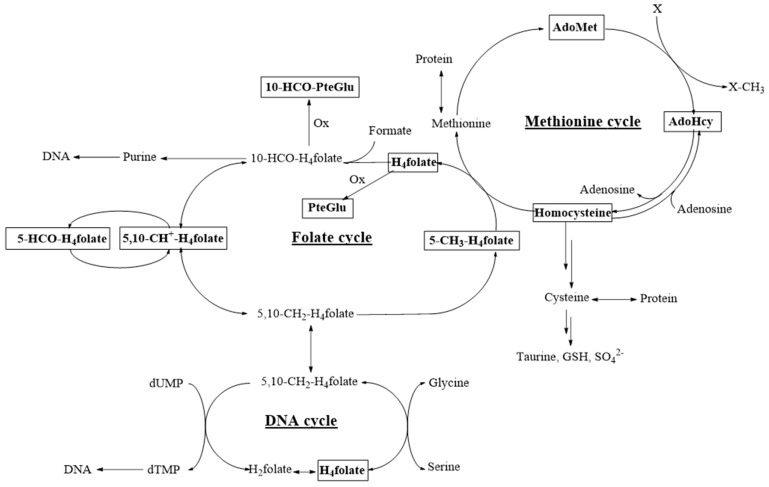
Analytes determined by stable isotope dilution assays coupled with liquid chromatography tandem mass spectrometry (SIDA-LC-MS/MS) in selected tissue samples. Ox: Oxidation, GSH: Glutathione, dTMP: Deoxythymidine monophosphate, dUMP: Deoxyuridine monophosphate. Analytes under study are framed in bold lines.

Therefore, our aim was to establish a mouse model using C57BL/6N mice to examine in detail and comprehensively the direct effects of a diet-induced folate deficiency on classical biomarkers like total homocysteine (tHcy) in plasma and erythrocyte folate and their correlation to tissue-specific folate status, vitamer distribution, and AdoMet or AdoHcy levels (Figure 1). This method could then complement methods more focused on unravelling the pathogenic mechanisms of folate deficiencies.

## 2. Materials and Methods 

### 2.1. Chemicals and Solutions

Chemicals, solutions, and calibrators for folate, AdoMet, and AdoHcy extraction in tissue and Hcy in plasma have been described recently [19]. Purity of AdoMet, AdoHcy, and Hcy was determined by quantitative nuclear magnetic resonance spectroscopy, whereas purity of folate standards was determined by liquid chromatography coupled with diode array detection (LC-DAD) [19]. Solutions for folate extraction in erythrocytes were prepared according to the method of Mönch et al. [29].

### 2.2. Animals and Ethics Approval 

For the folate deficiency study, male C57BL/6N mice were used. All animal procedures were approved by the Bavarian Animal Care and Use Committee (reference number 55.2-1-54-234-2013) and all experiments were performed in accordance with relevant guidelines and regulations. 

### 2.3. Study Design

Mice were bred and kept in animal facilities of the Technical University of Munich (TUM) Institute for Food and Health (ZIEL). All animals had free access to water. After 3 weeks weaning, mice were fed with a chemically defined standard chow (V1534 R/M-H; ssniff Spezialdiäten GmbH, Soest, Germany) for 1 week and subsequently divided into two groups with six animals each. Group 1 (*n* = 6) obtained folate-deficient diet (fdd) (ssniff Spezialdiäten GmbH, EF E15051 folic acid-deficient, ~0.06 mg/kg), whereas group 2 was fed with a defined control diet (cd) (ssniff Spezialdiäten GmbH, EF E15051 control, 16 mg/kg). The study was conducted for 12 weeks.

### 2.4. Sampling Procedure

For blood and tissue sampling, mice were sacrificed by isoflurane overdose. Blood was collected into ethylenediaminetetraacetic acid (EDTA)-coated tubes (Sarstedt, Nümbrecht, Germany) via cardiac puncture and centrifuged for 10 min at 1200× *g* and 4 °C to separate plasma. Plasma, erythrocytes, and tissues including brain, liver, kidney, and heart were harvested. Whole tissues (brain, heart, and right kidney) as well as the medial part of liver were flash-frozen in liquid nitrogen, and stored at −80 °C until use. Liver, heart, kidney, and brain were lyophilized for 24 h and subsequently homogenized with a micro pestle at room temperature to avoid condensation in the dried tissue powder. Aliquots of 10 mg of lyophilized liver, kidney, and heart and 20 mg of lyophilized brain were prepared for AdoMet and AdoHcy analyses in centrifuge tubes. Furthermore, 5 mg of freeze-dried liver, 10 mg of freeze-dried kidney, and 25 mg of lyophilized brain and heart were prepared in centrifuge tubes for folate extraction. All extracts were stored at −20 °C until LC-MS/MS measurement.

### 2.5. Folate in Liver, Brain, Heart, Kidney, and Erythrocytes 

The following were added to the tissue samples: 9.5 ng of [^2^H_4_]-5-CH_3_-H_4_folate, 6.1 ng of [^2^H_4_]-H_4_folate, 7.5 ng of [^2^H_4_]-5-HCO-H_4_folate, 1.7 ng of deuterated 10-formylfolate ([^2^H_4_]-10-HCO-PteGlu), and 2.1 ng of deuterated folic acid ([^2^H_4_]-PteGlu). After adding 2 mL of extraction buffer 2, samples were homogenized for 10 s with a TissueRuptor^®^ and placed in a chilled ultrasonic bath for lysis for 30 min. Aliquots of 150 µL of rat serum and 1 mL of chicken pancreas suspension were added, and the suspension was incubated for 4 h at 37 °C under constant agitation in a water bath. After heating for 4 min at 100 °C, samples were cooled in an ice bath and centrifuged at 2700× *g* and 4 °C for 20 min. Extraction of erythrocyte folate was carried out according to [29]. For each blood or tissue sample, one extraction was performed per mouse.

### 2.6. Solid-Phase Extraction (SPE)

All extracts were purified by SPE using a 12-port manifold (Merck, Darmstadt, Germany) equipped with Strata SAX cartridges (quaternary amine, 100 mg, 1 mL). The stationary phase was activated with two column volumes of methanol and two column volumes of equilibration buffer followed by the application of the respective tissue extracts. Afterwards, the cartridges were washed with two column volumes of equilibration buffer and subsequently dried by evaporation *in vacuo*. Elution was carried out with 0.5 mL eluting solution.

### 2.7. AdoMet and AdoHcy in Liver, Brain, Heart, and Kidney

Aliquoted tissue samples were placed in a freezing mixture (−80 °C) consisting of dry ice in acetone. Protein precipitation was carried out with 1.5 mL 100 mmol/L precipitation reagent dithiothreitol (DTT) in methanol/water (90/10 *v*/*v*) with 0.1% (*v*/*v*) formic acid), and 611 ng of [^2^H_3_]-AdoMet and 77.5 ng of [^2^H_4_]-AdoHcy were added to the lyophilized samples. The suspension was then homogenized for 30 s with a TissueRuptor^®^ (Qiagen, Hilden, Germany), subsequently incubated for 15 min at 4 °C, and placed in the freezing mixture again. After centrifugation at 2700× *g* and 4 °C for 20 min, the supernatant was removed and dried under a nitrogen flow. The pellet was re-suspended in 500 µL of dilution solvent consisting of 0.1% (*v*/*v*) formic acid in water/acetonitrile (98/2 *v/v*), vortex-mixed, and centrifuged for 3 min at 15,400× *g*. Aliquots of 200 µL of the supernatant were transferred into an autosampler vial for LC-MS/MS analysis. For each tissue sample, one extraction was performed per mouse.

### 2.8. tHcy in Plasma

Aliquots of 50 µL of mouse plasma were spiked with 30 ng of [^2^H_4_]-Hcy and vortex-mixed. After 15 min of equilibration, 50 µL of a 200 mmol/L aqueous DTT solution was pipetted into the plasma. Following further vortex-mixing, the sample was incubated for 30 min at ambient temperature and subsequently mixed with 0.35 mL of methanol for protein precipitation. After an additional 10 min in the freezer, the plasma was vortex-mixed, and the supernatant was removed by centrifugation at 15,400× *g* for 3 min and subsequently dried under nitrogen flow. The pellet was resuspended in 250 µL of dilution solvent (see Section 2.7), vortex-mixed, and centrifuged at 15,400× *g* for an additional 3 min. Two hundred microliters were transferred into an autosampler vial for LC-MS/MS analysis. For each plasma sample, one extraction was performed per mouse.

### 2.9. LC-MS/MS

Folates in tissue samples and erythrocytes were determined by means of LC-MS/MS (Finnigan Surveyor Plus high performance liquid chromatography (HPLC) System, Thermo Electron Corporation, Waltham, MA, USA; triple quadrupole TSQ quantum discovery mass spectrometer, Thermo Electron Corporation, Waltham, MA, USA). The vitamers were separated on a YMC Pack Pro C_18_ column (150 × 3 mm, 3 µm, YMC, Kyoto, Japan). The mobile phase for gradient elution consisted of 0.1% (*v*/*v*) aqueous formic acid (eluent A) and acetonitrile containing 0.1% (*v*/*v*) formic acid (eluent B) at a flow rate of 0.3 mL/min.

The system for Hcy, AdoMet, and AdoHcy measurement consisted of a Shimadzu Prominence LC-20A System (Shimadzu, Kyoto, Japan) and an API 4000 Q-Trap mass spectrometer (AB Sciex, Foster City, CA, USA). Analyte separation was carried out on a Phenomenex Gemini reversed phase column (110A 3u, 150 × 4.60 mm, Phenomenex, Aschaffenburg, Germany). The mobile phase for gradient elution consisted of 0.1% (*v*/*v*) aqueous formic acid (eluent A) and acetonitrile containing 0.1% (*v*/*v*) formic acid (eluent B) at a flow of 0.4 mL/min. 

Gradients and source parameters of the LC-MS instrument for tissue, plasma samples [19], and erythrocytes [29] have been published earlier.

### 2.10. Data Analysis

Data analysis was carried out using Xcalibur Software ver. 2.0 (Thermo Scientific, Waltham, MA, USA) and Analyst Software ver. 1.6.2 (AB Sciex, Foster City, CA, USA). NMR spectra were processed using TopSpin 3.0 software (Bruker, Billerica, MA, USA). Two-sided *t*-test (*p* = 0.05) for significance was performed with Microsoft Excel 2013 (Microsoft, Redmond, WA, USA).

## 3. Results

### 3.1. Body Weight

**Figure 2:**
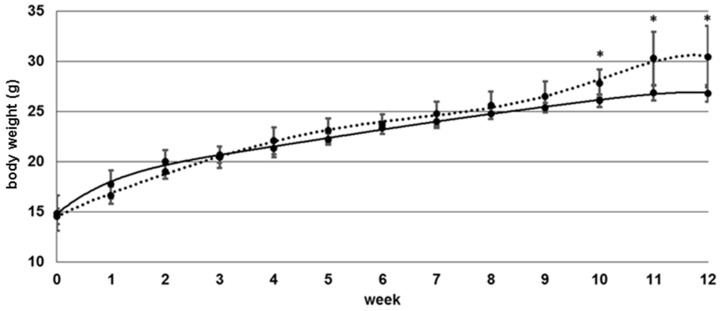
Body weight of controls (*n* = 6, dashed line) and mice fed a folate-deficient diet (*n* = 6, straight line), * significant difference (*p* < 0.05).

Control vs. folate deficient group showed no significantly (*p* > 0.05) different body weight during the study period within week 1–10 (Figure 2).

From week 10–12 we observed a higher mean body weight in the control group (*p* < 0.05) with a maximum at 12 weeks. 

### 3.2. Analysis of tHcy and Folates in Plasma and Erythrocytes

**Figure 3:**
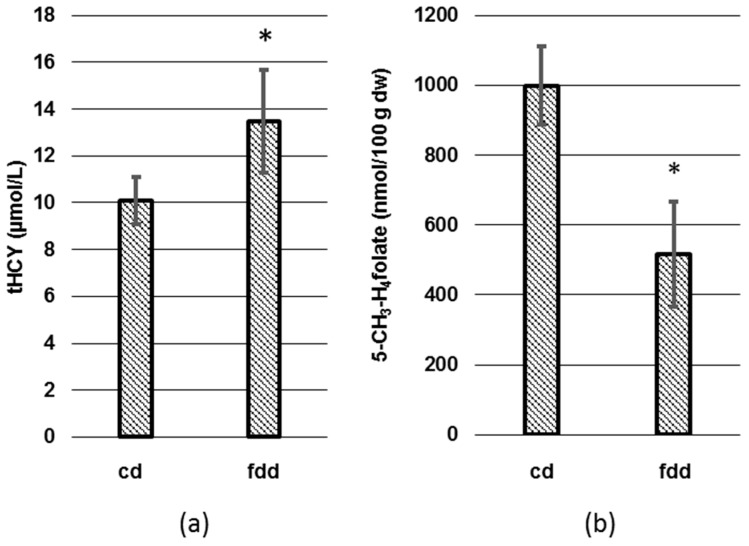
Total homocysteine (tHcy) (**a**) in plasma and 5-CH_3_-H_4_folate in lyophilized erythrocytes (**b**). dw: Dry weight, cd: Control diet (*n* = 6), fdd: Folate-deficient diet (*n* = 6), * significant difference (*p* < 0.05).

In a first experiment, biomarkers of folate deficiency were determined in plasma and erythrocytes (Figure 3). 

The control group showed tHcy levels of 10.1 ± 1 µmol/L, whereas elevated tHcy levels of 13.5 ± 2.2 µmol/L were found in the folate-deficient group after 12 weeks, corresponding to a relative increase of 33.7% (*p* < 0.05). In contrast, we observed in the deficient group showing 518 ± 151 nmol 5-CH_3_-H_4_folate/100 g dw a 48% (*p* < 0.05) lower erythrocyte 5-CH_3_-H_4_folate level than in the control group revealing 999 ± 112 nmol 5-CH_3_-H_4_folate/100 g dw. Taking into account the water content of erythrocytes (68%), concentrations in fresh erythrocytes were 3219 ± 353 nmol/L and 1677 ± 482 nmol/L, respectively. This is in rather good agreement with Leamon et al. [33], who observed an initial erythrocyte folate concentration of 4329 nmol/L in BALB/c mice that was reduced by 77% to 1000 nmol/L after 12 weeks of folate-deficient diet.

Because 5-CH_3_-H_4_folate was the only vitamer detected in lyophilized erythrocytes, the term “erythrocyte folate” is used in the following context and refers to the 5-CH_3_-H_4_folate concentration.

### 3.3. Determination of Folate Patterns and Total Folate Content in Tissues

**Figure 4:**
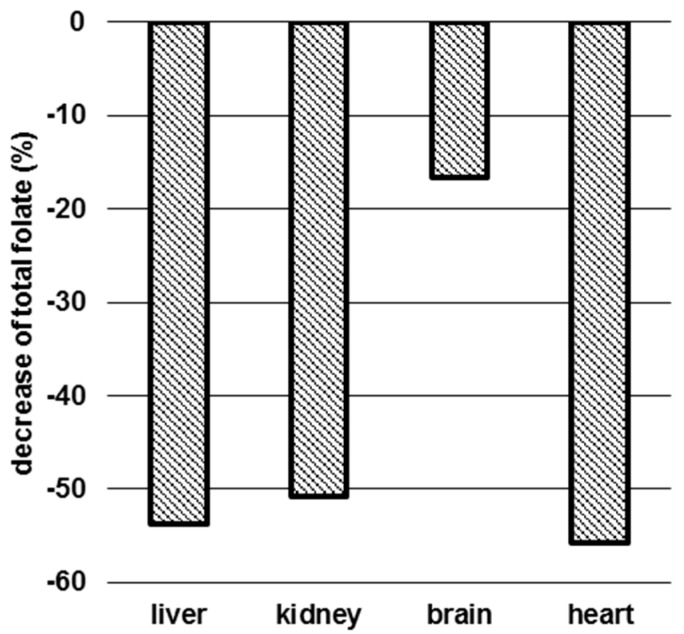
Percental decrease of total tissue folate in folate-deficient mice after 12 weeks. cd: Control diet (*n* = 6), fdd: Folate-deficient diet (*n* = 6).

Total folate contents of 135, 54.1, 3.52, and 4.44 nmol calculated as PteGlu/g dw were found in liver, kidney, brain, and heart of controls, respectively, whereas 62.4, 26.7, 2.94, and 1.97 nmol PteGlu/g dw were determined in tissues of folate-deficient mice, respectively. In brain, we observed a decrease in total folate of 17%. Folate contents in liver, kidney, and heart of deficient mice were even more depleted, being 54%, 51%, and 56%, respectively, lower than in mice fed a control diet for 12 weeks (Figure 4) and, thus, similar to the decrease of 5-CH_3_-H_4_folate in erythrocytes.

**Figure 5:**
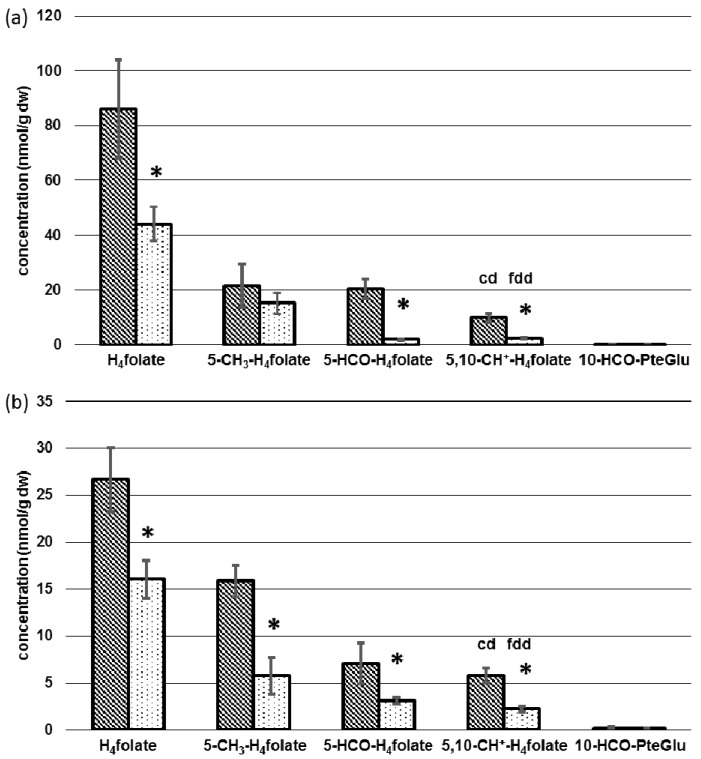
Tissue-specific concentrations of folate vitamers depending on diet in (**a**) liver and (**b**) kidney. dw: Dry weight, cd: Control diet (*n* = 6), fdd: Folate-deficient diet (*n* = 6), * significant difference (*p* < 0.05).

Concentrations of each folate vitamer in liver and kidney depending on diet are depicted in Figure 5a,b.

H_4_folate constitutes the main folate vitamer in liver of controls and folate-deficient mice with 86.1 ± 18.1 nmol/g dw and 44.0 ± 6.21 nmol/g dw (−49%), respectively (*p* < 0.05). A stronger depletion of 91% was observed for 5-HCO-H_4_folat with 20.4 ± 3.47 and 1.86 ± 0.32 nmol/g dw (*p* < 0.05) as well as 77% for 5,10-CH^+^-H_4_folate with 9.88 ± 1.54 and 2.28 ± 0.52 nmol/g dw (*p* < 0.05) for controls and folate-deficient mice, respectively. 10-HCO-PteGlu was detectable only in traces (<0.3 nmol/g dw). Compared to the aforementioned vitamers, no significant difference was found for 5-CH_3_-H_4_folate, with 15.2 ± 3.83 nmol/g dw (fdd) and 21.6 ± 7.96 nmol/g dw (cd) (*p* > 0.05). 

Concentrations of folate vitamers in kidney (Figure 5b) were lower than in liver. As in liver, H_4_folate constitutes the main vitamer. After 12 weeks, 26.6 ± 3.41 nmol/g and 16.0 ± 2.03 nmol/g dw (−40%) (*p* < 0.05) were found for controls and folate-deficient mice, respectively. Whereas liver 5-CH_3_-H_4_folate remained almost constant, a 64% lower level of 5.77 ± 1.96 nmol/g dw was observed in kidney of folate-deficient mice compared to 15.9 ± 1.70 in controls (*p* < 0.05). Concentrations of 5-HCO-H_4_folate and 5,10-CH^+^-H_4_folate were 56% (7.10 ± 2.13 dw and 3.11± 0.37 nmol/g dw) and 62% (5.81 ± 0.81 dw and 2.23 ± 0.28 nmol/g dw) lower in folate-deficient mice after 12 weeks (*p* < 0.05). 10-HCO-PteGlu was detectable only in traces (<0.03 nmol/g dw).

**Figure 6:**
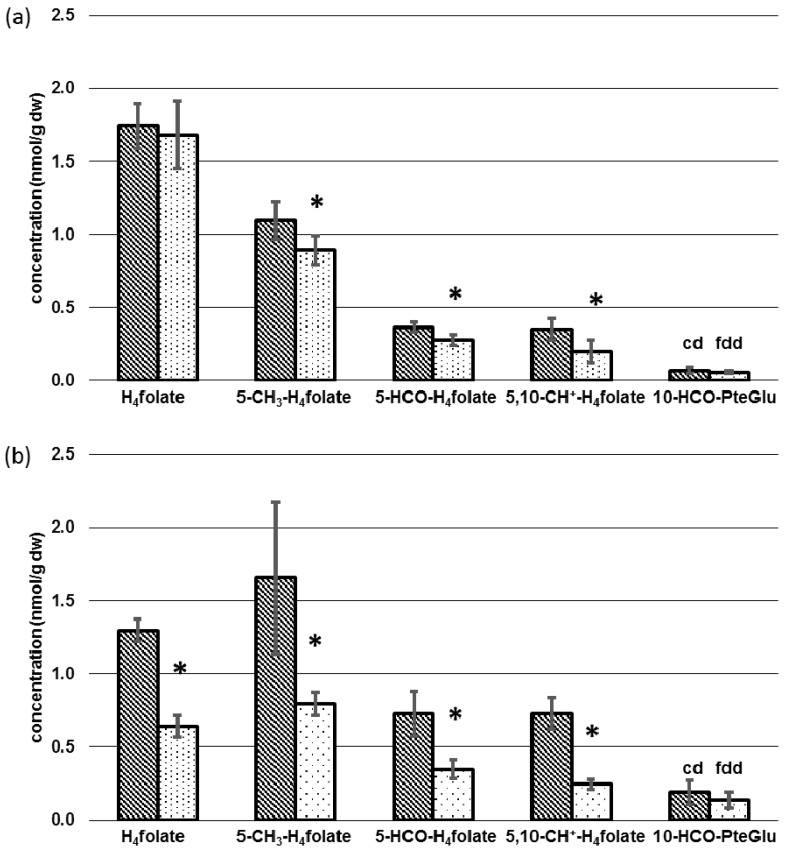
Tissue-specific concentrations of folate vitamers depending on diet in (**a**) brain and (**b**) heart. dw: Dry weight, cd: Control diet (*n* = 6), fdd: Folate-deficient diet (*n* = 6), * significant difference (*p* < 0.05).

Compared to liver and kidney, folate contents in brain and heart (Figure 6a,b) were about a factor of 35 or 15 lower, respectively. 

Total folate status in brain of depleted mice remained almost constant. For H_4_folate, no significant difference was observed for both groups, with 1.68 ± 0.23 nmol/g dw (fdd) and 1.74 ± 0.15 nmol/g dw (cd) (*p* > 0.05). Lower brain folate in deficient mice could be ascribed to significant lower levels (*p* < 0.05) of 5-CH_3_-H_4_folate, 5-HCO-H_4_folate, and 5,10-CH^+^-H_4_folate with 1.09 ± 0.13 nmol/g dw vs. 0.89 ± 0.10 nmol/g dw (−18%), 0.36 ± 0.03 nmol/g dw vs. 0.28 ± 0.04 nmol/g dw (−22%), and 0.35 ± 0.07 nmol/g dw vs. 0.20 ± 0.07 nmol/g dw (−43%) for controls and folate-deficient mice, respectively. 

In contrast to other tissues 5-CH_3_-H_4_folate constitutes the main vitamer in heart. Compared to the controls (with 1.66 ± 0.52 nmol/g dw), deficient mice (with 0.80 ± 0.08 nmol/g dw) revealed a 52% lower 5-CH_3_-H_4_folate level. H_4_folate, 5-HCO-H_4_folate, and 5,10-CH^+^-H_4_folate levels were 1.30 ± 0.08 nmol/g dw vs. 0.64 ± 0.07 nmol/g dw (−51%), 0.73 ± 0.15 nmol/g dw vs. 0.35 ± 0.07 nmol/g dw (−52%), and 0.73 ± 0.11 nmol/g dw vs. 0.24 ± 0.03 nmol/g dw (−67%), for controls and folate-deficient mice, respectively (*p* < 0.05). Neither brain nor heart showed significant lower 10-HCO-PteGlu levels in folate-deficient mice. Levels remained low with <0.1 nmol/g dw in brain and <0.2 nmol/g dw in heart. 

### 3.4. Determination of AdoMet and AdoHcy in Tissue 

**Figure 7:**
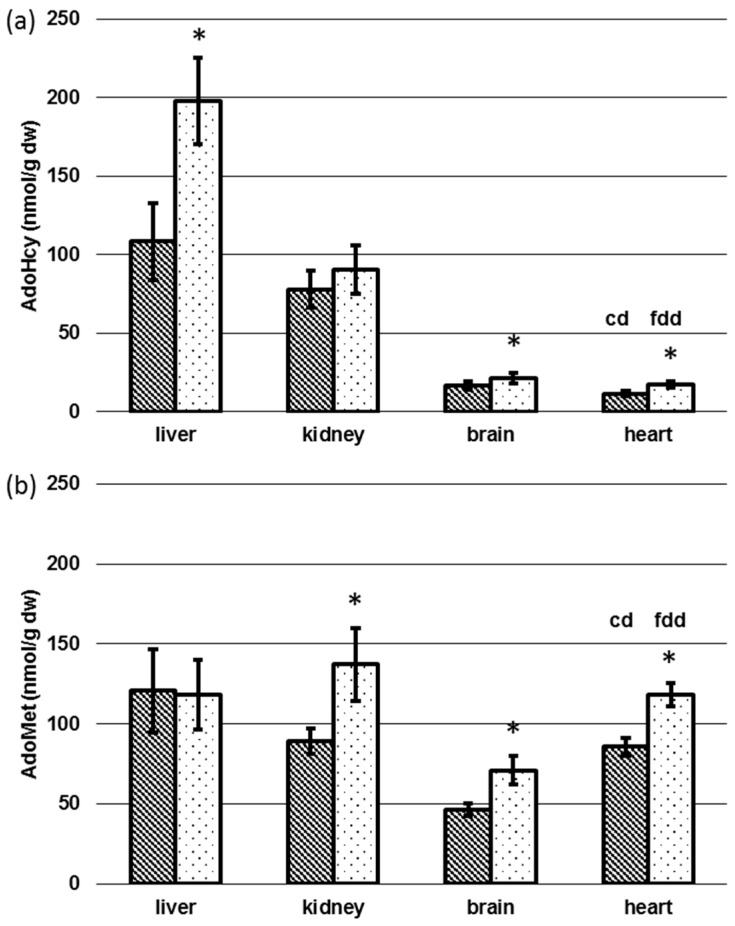
Tissue-specific concentrations of *S*-adenosylhomocysteine (AdoHcy) (**a**) and *S*-adenosylmethionine (AdoMet) (**b**) depending on diet. dw: dry weight, cd: Control diet (*n* = 6), fdd: Folate-deficient diet (*n* = 6), * significant difference (*p* < 0.05).

In addition to biomarkers of folate deficiency in blood and folate status in tissues, we determined AdoMet and AdoHcy in the same organs to examine the impact of dietary induced folate deficiency on methionine cycle. Figure 7 shows the tissue-specific levels of AdoMet and AdoHcy depending on diet.

In controls, liver and kidney contained the highest AdoHcy content (A), with 108 ± 24.6 and 77.9 ± 11.5 nmol/g dw, when compared to brain and heart, with 16.4 ± 2.65 and 11.4 ± 1.79 nmol/g dw, respectively. Folate deficiency caused dramatically elevated hepatic AdoHcy levels, with 198 ± 27.5 nmol/g dw (+83%) (*p* < 0.05), whereas the discrepancy was lower in kidney, brain, and heart of folate-deficient mice, with 90.3 ± 15.4 nmol/g dw (+16%, *p* > 0.05), 21.2 ± 3.21 nmol/g dw (+29%, *p* < 0.05), and 17.1 ± 2.20 nmol/g dw (+50%, *p* < 0.05), respectively.

**Figure 8:**
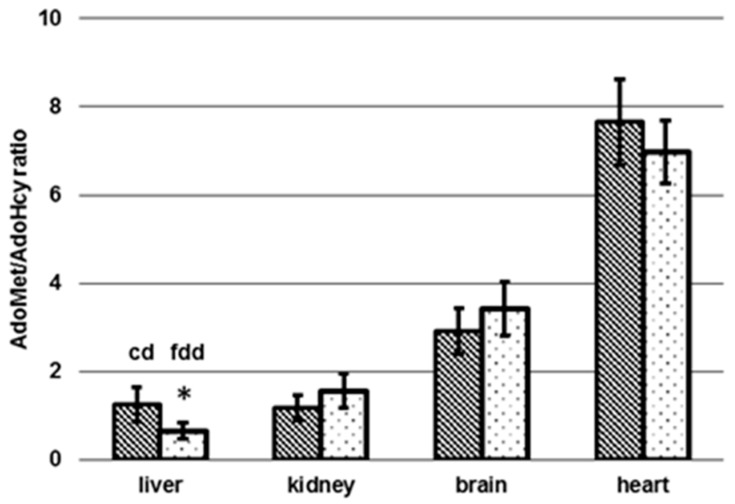
Tissue-specific AdoMet/AdoHcy ratio depending on diet. cd: Control diet (*n* = 6), fdd: Folate-deficient diet (*n* = 6), * significant difference (*p* < 0.05).

In the controls, concentrations of AdoMet in liver and kidney, with 121 ± 25.9 nmol/g dw and 89.3 ± 7.93 nmol/g dw, respectively, were similar to the respective AdoHcy levels. In contrast, AdoMet in heart, with 85.7 ± 5.41 nmol/g dw, was comparable to AdoMet in kidney, and AdoMet in brain, with 46.5 ± 3.81 nmol/g dw, was about half the concentration observed in kidney. Dietary induced folate deficiency led to no significant change of AdoMet levels in hepatic tissue, with 118 ± 21.7 nmol/g dw (*p* > 0.05). For renal tissue, brain, and heart we observed significantly (*p* < 0.05) elevated AdoMet concentrations in fdd mice, with 137 ± 22.1 nmol/g dw (+53%), 71.0 ± 8.69 nmol/g dw (+53%), and 118 ± 7.16 nmol/g dw (+38%), respectively. As depicted in Figure 8, significant differences in the methylation capacity (AdoMet/AdoHcy ratio) were obtained for liver (1.25 ± 0.40 (cd) 0.65 ± 0.18 (fdd)) (*p* < 0.05), whereas AdoMet/AdoHcy remained almost constant in kidney, brain, and heart.

## 4. Discussion

### 4.1. Body Mass and Erythrocyte Folate Status

Mice were weighed weekly and significantly lower weight gain was observed for folate-deficient mice after 10 weeks. In a folate deficiency study with rats [8], a significant difference was determined even after 3 weeks. In the latter study, hematocrit decreased dramatically from 40 to 20% after 4 to 6 weeks. Tissue-specific folate levels in liver and kidney decreased by more than 50% within 2 weeks.

Dietary induced folate deficiency in C57BL/6N mice caused a ~50% reduction of folate levels in erythrocytes, liver, kidney, and heart. Therefore, erythrocyte folate reflects folate status of tissue stores except brain, where only a marginal decrease of 17% was observed. These findings were consistent with the investigation of Wu et al. [34], showing that erythrocyte folate correlates well with hepatic folate in humans. Because of the low blood specimen obtained from C57BL/6N mice, no further investigations were made on hematocrit and possibly limited erythropoiesis.

### 4.2. Tissue-Specific Changes of Metabolic Profiles

Compared to liver and kidney, total folate in brain and heart was 35- and 15-fold lower, respectively, underlining their dependency on these folate-accumulating tissues. Because of its high hepatic concentration, H_4_folate can be regarded as reserve folate. In fact, H_4_folate constitutes a better substrate for pteroylpolyglutamate synthetase than substituted vitamers, leading to a more effective transformation to storable long-chain polyglutamates [35,36].

In kidney, brain, and heart a general depletion of all folate vitamers was observed, whereas 5-CH_3_-H_4_folate remained almost constant in liver. This might be due to activation of methylenetetrahydrofolate reductase (MTHFR) by excessive hepatic AdoHcy [26] leading to a reduction of 5-HCO-H_4_folate, 5,10-CH^+^-H_4_folate, and especially H_4_folate as the main vitamer in hepatic tissue. Because of the strong reduction of 5-CH_3_-H_4_folate in kidney (−64%) and heart (−52%), we assume that this vitamer might be largely retained by hepatic cells after methylation of polyglutamylated H_4_folate to ensure maintenance of essential metabolic processes. Almost 90% of methyl-groups deriving from AdoMet are used by hepatic tissue for creatine synthesis [22,23,24], thus unequivocally leading to an increase in AdoHcy. Methionine synthase utilizing 5-CH_3_-H_4_folate is responsible for recycling and remethylation of Hcy. In liver and possibly kidney of humans and the liver of rodents, an alternative pathway using BHMT coexists [37]. Nevertheless, AdoHcy hydrolase reaction, cleaving AdoHcy to adenosine and Hcy, is reversible, and increasing amounts of AdoHcy of up to 83% in liver might lead to inhibition of methionine synthase and BHMT [26]. This assumption is supported by the significant (*p* < 0.05) inversion of the hepatic AdoMet/AdoHcy ratio representing a decrease of intracellular methylation capacity. Excessive 5-CH_3_-H_4_folate is released into the bile, thereby entering the enterohepatic cycle [38]. Following reabsorption in the small intestine, 10–20% of the substituted folates are accumulated by hepatic tissue after the first passage [39]. The remaining part is distributed to other organs at the expense of hepatic folate expressed by a decrease of 44 nmol H_4_folate/g dw. 

For the supply of AdoMet, an alternative metabolic pathway exists exclusively in liver. The transformation of dietary methionine is catalyzed by methionine adenosyltransferase III [40], which is not inhibited by high cellular AdoHcy concentrations.

Concentrations of 5-HCO-H_4_folate, which can be regarded as a storage form of formylated folates [41], declined significantly in all tissue samples under study. This could be due to interconversion of 5-HCO-H_4_folate and 5,10-CH^+^-H_4_folate to 10-HCO-H_4_folate to maintain essential purine synthesis. 10-HCO-H_4_folate is unstable in acidic mobile phases used for LC-MS/MS analysis of folates [42] because of its interconversion to 5,10-CH^+^-H_4_folate [43]. Moreover, 5-HCO-H_4_folate is formed after heating of 10-HCO-H_4_folate [43,44]. Hence, the latter vitamer was not accessible by our method. 

The lower discrepancy between controls and folate-deficient mice for brain folate might be due to the presence of respective folate receptors, a high-affinity uptake system for folate located in the choroid plexus, maintaining folate transport into the brain against the plasma/cerebrospinal fluid (CSF)-concentration gradient even at low folate status [9,45].

In kidney, brain, and heart, we noticed a significant increase of AdoMet as well as a lower significant increase of AdoHcy in brain and heart, resulting in identical AdoMet/AdoHcy ratios (Figure 8) (*p* > 0.05) in folate-deficient mice compared to controls (Figure 7a,b). As both diets contain the same methionine concentration a higher feed and, therefore, methionine intake in the folate-deficient group could be a possible explanation [40]. However, feed uptake and methionine levels were not determined.

### 4.3. Changes of tHcy in Plasma

Liver, kidney, and pancreas express the highest AdoHcy hydrolase activity [46]. Because of the reversibility of the hydrolase reaction, increasing concentrations of adenosine and homocysteine lead to a formation of AdoHcy [47]. In contrast, cystathionine-β-synthase (CBS) is activated by AdoHcy and removes excessive Hcy by the transsulfuration pathway in liver and kidney [48]. Therefore, tHcy in plasma increased by only 33.7%, being neither proportional to the 50% decrease of erythrocyte folate nor to the dramatic increase of AdoHcy in liver. A major difference between humans and mice is the extent of urinary excreted Hcy. Renal absorption of Hcy is almost quantitative (99%) in humans, whereas 38% of tHcy are excreted by mice. Therefore, mice could be less susceptible to high tHcy and toxic effects [49]. 

Plasma tHcy is discussed as a potential biomarker for an intracellularly reduced methylation capacity [13]. This assumption implies that Hcy represents a transport form of cellular AdoHcy which enables the cell to release excessive AdoHcy to maintain methylation capacity [26]. Nevertheless, it remains unclear from which tissue and to what extent AdoMet or AdoHcy are released by cells [50]. In fact AdoMet and AdoHcy levels are by far higher in cells than in the circulation [26], which is not the case for Hcy [46].

## 5. Conclusions

We herein present the first minimized multi SIDA-LC-MS/MS approach for detailed screening of six folate vitamers, AdoMet, and AdoHcy in lyophilized tissue samples as well as tHcy in plasma and erythrocyte folate of C57BL/6N mice (folate-deficient/control). The use of isotopologic internal standards enables precise quantitation of all metabolites under study and, thus, evaluation of direct metabolic impact of folate deficiency on all relevant markers and their interrelations. A model for alterations in hepatic one-carbon metabolism was successfully developed. 

The decrease of erythrocyte folate correlated with the decrease of total folate levels in liver, kidney, and heart. Therefore, erythrocyte folate represents a powerful diagnostic mean to determine folate depletion in these organs. In contrast, brain is less susceptible to dietary induced folate deficiency, even at 50% lower folate status. H_4_folate is accumulated primarily by hepatocytes and represents the main vitamer in all organs except heart (5-CH_3_-H_4_folate) followed by 5-CH_3_-H_4_folate (heart: H_4_folate), 5-HCO-H_4_folate, and 5,10-CH^+^-H_4_folate. THcy levels increased by 33.7% in plasma of folate-deficient mice and may be a product of intracellular AdoHcy detoxification, originating mainly from hepatocytes (+83% AdoHcy), to maintain cellular methylation capacity. Compared to erythrocyte folate, tHcy is a less specific marker because no direct correlation was found to tissue-specific levels of folate, AdoHcy, or AdoMet.

The successfully developed method presented here may be helpful for studying mechanisms of pathogenesis due to folate deficiency when combined with other markers of hypomethylation. Current methods often lack a comprehensive view of one-carbon metabolites [51].
